# Land Use Changes and GHG Emissions from Tropical Forest Conversion by Oil Palm Plantations in Riau Province, Indonesia

**DOI:** 10.1371/journal.pone.0070323

**Published:** 2013-07-30

**Authors:** Fatwa Ramdani, Masateru Hino

**Affiliations:** Geoenvironment, Institute of Geography, Earth Science Department, Graduate School of Science, Tohoku University, Sendai, Japan; University of Massachusetts, United States of America

## Abstract

Increasing prices and demand for biofuel and cooking oil from importer countries have caused a remarkable expansion of oil palm plantations in Indonesia. In this paper, we attempt to monitor the expansion of oil palm plantations on peat land and in tropical forests. We measure the GHG emissions from the land conversion activities at provincial scale. Using Landsat images from three different periods (1990s, 2000s and 2012), we classified LULC of the Riau Province, which is the largest oil palm producing region in Indonesia. A hybrid method of integration, generated by combining automatic processing and manual analysis, yields the best results. We found that the tropical rainforest cover decreased from ∼63% in the 1990s to ∼37% in the 2000s. By 2012, the remaining tropical rainforest cover was only ∼22%. From the 1990s to the 2000s, conversion of forests and peat lands was the primary source of emissions, total CO_2_ emitted to the atmosphere was estimated at ∼26.6 million tCO_2_.y^-1^, with 40.62% and 59.38% of the emissions from conversion of peat lands and forests, respectively. Between 2000 and 2012, the total CO_2_ emitted to the atmosphere was estimated at ∼5.2 million tCO_2_. y^-1^, with 69.94% and 27.62% of the emissions from converted peat lands and converted forests, respectively. The results show that in the Riau Province, the oil palm industry boomed in the period from 1990 to 2000, with transformation of tropical forest and peat land as the primary source of emissions. The decrease of CO_2_ emissions in the period from 2000 to 2012 is possibly due to the enforcement of a moratorium on deforestation.

## Introduction

Oil palm plantations in Sumatera and Kalimantan produce ∼50% of oil palm worldwide, and in the near future Indonesia’s government plans to double production by expansion of plantations on the Eastern side of the country [1]. Higher prices and demand for biofuel and cooking oils, drive tropical forest conversion into oil palm plantations [2]. From the first introduction up to 2011, Indonesia’s oil palm estates reached 8.7 million hectares [3]. This remarkable increase in demand for bio-energy in the world played a major role in Indonesia’s natural tropical forest conversion. Indonesian oil palm production is mostly exported to foreign countries. The total exports of Indonesia’s oil palm in the year 2011 increased to U.S. $ 19.38 billion from U.S. $ 4.34 billion in the year 2005 [3].

Oil palm is a crop commodity, which plays an important role in economic activities in Indonesia. Indonesia is the largest producer of palm oil, with 9 million tonnes produced in 2010. Together with Malaysia, this accounted for 84.36% of worldwide production in the period 2000-2010 [4]. In 2011, five major Indonesian oil palm importing countries were India (50.54%), Malaysia (14.77%), Singapore (7.51%), the Netherlands (7.16%), and Italy (5.78%). India’s import reached 4.26 million tonnes of the total volume of Indonesia’s CPO exports valued, equal to US$ 4.46 billion [3]. In India, vegetable oil is most widely used for domestic use, industrial food processing plants, restaurants and hotels. This oil is used for frying foods and making cakes. Ninety % of the palm oil is used in food products such as margarine, shortening, and vegetable cooking oil, while the remaining 10% is used by various industries [5].

The conversion of tropical forest and peatlands leads to substantial GHG emission by LULC changes [6]. Indonesia is a top GHG emitter, largely due to forest conversion [7]. Other main factors in the GHG emission are the peatland conversion to oil palm plantations, and the increasing number of industrial forests and illegal logging operations, which may finally lead to increase in the risk of forest-burning. Draining of peatlands due to widespread forest industrial concessions and illegal logging activities. The construction of canals and drainages lead to increased risk of fire [8], [9].

However, at provincial level, emissions are uncertain, since most research focuses on global and national scale. Furthermore, emissions from oil palm plantations are poorly recorded in Indonesia. Therefore, quantifying the contribution of oil palm plantations to global GHG emissions and understanding the expansion of oil palm production areas are important.

Most studies have focused on identifying land source emissions at regional scale, for example, Koh et al. [10] quantify the expansion of oil palm plantations in tropical peatlands in the 2000s in the Peninsular Malaysia, Sumatera, and Borneo. Gibbs et al. [11] analyse the agricultural expansion across the tropics using a rich satellite library. Carlson et al. [12] analyse Kalimantan’s forest conversion for oil palm plantations from 1990 to 2010 using Landsat satellite images and the estimated carbon flux from the Kalimantan plantations. Finally Canadell et al. [13] estimated that CO_2_ emission from peatland drainage in Southeast Asia is contributing the equivalent of 1.3% to 3.1% of current global CO_2_ emissions from the combustion of fossil fuel.

Given that there is no prior scientific documentation at provincial level, we investigated the patterns in the expansion of oil palm plantations, land sources for oil palm production, and GHG emissions resulting from tropical forest conversion in the Riau Province, Indonesia.

## Materials and Methods

### Study Area

The Riau Province study area (∼8.9 million ha) lies at 2–91 m above sea level (asl). This province has a tropical climate, with an average rainfall ranging from 1,000-3,000 mm per year, with a dry season (April–September) and a rainy season (October–March). The Riau Province is located between 01° 05’ 00” South latitude - 02° 25’ 00” North latitude, and at between 100° 00’ 00”- 105° 05’ 00” East longitude. Regional Indonesian residents have diverse ethnicities (e.g. Melayu, Minangkabau, Jawa, Batak, Bugis/Makassar, Tionghoa, and Arab), including state-sponsored and independent transmigrants. The population density in 2010 was 64 people/km^2^, concentrated in the provincial capital city Pekan Baru.

### Data

The analysis of oil palm plantations in this study was derived from two satellite images: Landsat 5 TM & Landsat 7 ETM+ from http://glovis.usgs.gov/. The resolution of enhanced thematic mapper plus (ETM+) has a spatial resolution of 30 m for blue, green, red, near IR, SWIR, and mid-IR wavelength. The resolution of the thematic mapper (TM) is the same for the blue to mid-IR reflectance bands. For the Riau Province, we used 14 scenes in 1990s (1990–1993) and 2000s (2000–2003), while in 2012 we used 22 scenes, because there were gaps in the image acquisition in 2012. To fill the gaps we needed a minimum of two images which can serve as a filler and one image that can act as the main image to be filled, with the same month/seasons of acquisition. Fortunately, the cloud cover of our images varies only between 11 and 22%.

### Land Cover Classification

The main software used for analysing the data is ENVI. This software has a powerful image processing operation capability. The raw data were atmospherically corrected for reflectance, and for slope of the soil line derived from a regression analysis of the reflectance values contained in the near infrared band by those contained in the red band. This step was employed to separate green vegetation from soil background. WDRVI (Wide Dynamic Range Vegetation Index) transformation was done in single-era mosaicked images for fractional vegetation cover analysis (i.e. soil exposed, non-photosynthetic vegetation, and photosynthetic vegetation). A decision-tree algorithm was employed to detect the transformation from tropical rainforest into oil palm plantations. There are eight steps comprised in the decision-tree classification method: (i) atmospheric correction into top atmospheric reflectance from raw DN data; (ii) WDRVI transformation; (iii) enhanced Lee adaptive filter [14] to decrease the high spatial frequency of the images; (iv) classification of land use land cover based on supervised method (user-selected samples), unique type of oil palm appeareance in satellite images are represented in gridded pattern of roads networks; (v) segmentation images based on supervised image classification; (vi) majority and clump analysis; (vii) reclassification of missclassified class (i.e. oil palm plantation area <10 ha into the forest; (viii) convert raster land use land cover classification into a vector as manually digitized from classification result.

A hybrid integration method, on the basis of digital datasets generated by combining the automatic processing and manual analysis yielded the best results, especially when unique gridded patterns indicating variables oil palm development (land clearing) and gridded road network in new planted, young and mature oil palm trees are included as one class.

Seven land use/land cover classes were produced from the classification: (i) cultivated (consist of land for growing crop by farmers and traditional local peoples); (ii) oil palm plantation (consist of land clearing, new planted, young and mature trees); (iii) forest (consist of primary, secondary, wetland, and mangrove); (iv) settlement (consist of urban and rural areas); (v) other plantation (rubber and productive forest plantation); (vi) oil and gas field; (vii) water body (consist of river and lake).

Fortunately, our study areas are relatively flat, so we do not have to use the slope and aspect data to correctly classify brights or dark pixels in the images. Masking was done only for the cloud - cover and its shadow.

### Land Cover Classification Validation

Converted vector data were transformed into a KML/KMZ format to make data available for display and assessment of land cover validation done in Google Earth. 164 points (1990s: n = 52; 2000s: n = 57; 2012: n = 55) were selected randomly from each classification result. Points were then superimposed and a visual assessment was done. The extensive field work survey was conducted from May 5 to May 23, 2012 to verify points using the global positioning system (GPS). Next, we generated confusion matrices to calculate Kappa coefficients (k) and to derive overall accuracy. Kappa coefficient (k) of validations points is 0.75 and overall accuracy assessment is 0.87.

### Analysis of Oil Palm Expansion in Different Land Types

To assess the expansion of oil palm plantations in the Riau Province, we obtained GIS datasets from the Riau Province Government (i.e. Riau Province Spatial Plan – RTRWP/Rencana Tata Ruang Wilayah Propinsi). Moratorium (intact forest, peatlands and mangrove) GIS datasets were obtained from the Minister of Forestry. Data on administrative boundaries were derived from the National Geo-spatial Information Beaureau (BIG - Badan Informasi Geo-spasial). Datasets on oil palm plantation and land clearing were superimposed on the moratorium dataset to understand the role of the moratorium policy and the genesis of land of oil palm plantations.

### GHG Emission Measurement

The assumption used is that oil palm plantations will produce emissions if located on land that was previously used by the sectors or types of land use that have larger carbon stocks. The 1990s are used to describe the initial state. By identifying the extent of land use change at provincial level from 1990s to 2000s and from 2000s to 2012, and associating the change with the standard carbon stock value owned by each land use (Table 1), the carbondioxide emission can then be measured. The next step is to measure the amount of CO_2_ released by each land use class. The amount of CO_2_ emissions is obtained by multiplication of the carbon loss value with the molecular weight ratio of carbon dioxide to carbon, *i.e.* 3.67 [15]. The equations to calculate the CO_2_ emission from GIS datasets are as follows:
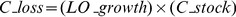
(1)

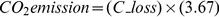
(2)Where:

**Table 1:** Standard carbon stock in Indonesia [16].

LULC	Carbon stock (Mg.ha^-1^)
**Cultivated**	23.17
**Forest**	200^1^
**Peatlands**	4.14
**Other plantation**	34.96^2^

1 derived from the average value of primary, secondary, and logged-over-forest.

2 derived from the average value of plantation in general and rubber plantation.

1 Mg = 1 ton.

*C_loss* = Carbon loss of land which used to be as peatlands or forest.

*LO_growth* = growth of land occupation by oil palm plantations in two different periods (ha) on land which used to be peatlands or forest.

*C_stock* = Carbon stock (Mg.ha^−1^) in land which used to be peatlands or forest), see Table 1.

## Results and Discussion

### Oil Palm Development and Land Sources

**Figure 1:**
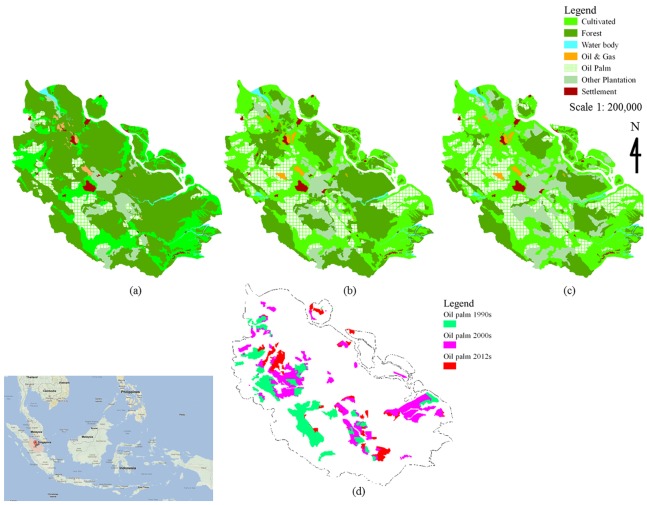
Study area and oil palm development. (a)1990s as the initial state; (b) 2000s; (c) 2012; (d) changes detected for oil palm development.

**Figure 2:**
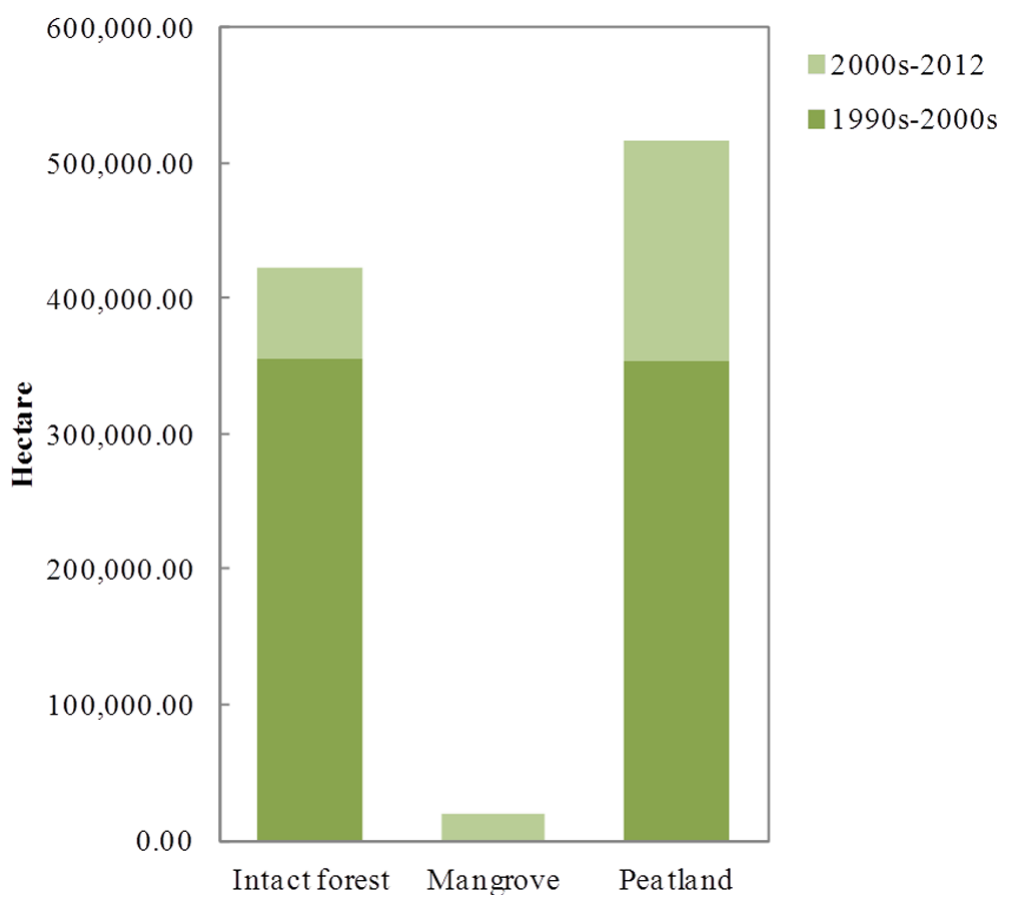
Land source of oil palm development.

Areas cleared for oil palm plantation in the Riau Province were obtained from Landsat satellite images (pixel resolution 30 m, n = 36 scenes) in 1990s, 2000s, and 2012. In this province, land clearing for oil palm plantation was first observed in the 1988 on the North-West and South-East part. In the 1990s, oil palm plantations covered ∼0.7 million ha (∼8% of the total provincial area). From 1990s to 2000s, the plantations expanded to occupy ∼1.4 million ha (∼16% of the total provincial area), and by 2012, they covered ∼1.6 million ha (∼20% of the total provincial area, see Fig. 1). Changes in policy and world market demand seemingly played a key role in this expansion [17], [18]. Between 1990 and 2000s, intact forest and peatlands were the primary land areas used for conversion into oil palm plantations. Meanwhile between 2000s and 2012 period, 28% of oil palm plantation was based on mineral soil of intact and logged forest, 70% from peatlands and 2% from converted mangrove (Fig. 2). Oil palm expansion in the Riau Province was controlled by lease allocation and National & Provincial Government policies, and influenced by market demand. By 2010, there were 139 oil palm companies in the Riau Province, consisting of two public companies (i.e. PT. Perkebunan Nusantara V & VI) and 137 private multinational companies [19]. The years between 1996 and 2002 were the period of privatization and cooperation, which corresponded with the reformation era following the termination of President Soeharto’s governance. During this period, the governor of the province acquired more powers to encourage economic growth by the development of oil palm plantations. A new policy was established and the governor could grant permission for land utilization up to 10,000 ha to private companies, from only 200 ha in the period between 1993 and 1996 (deregulation period). Permits for land utilization of areas more than 20,000 ha could only be issued by the Ministry of Forestry and Plantation in Jakarta. From 2002 up to recently, even regents could grant permission for land allocation up to 1,000 ha [20]. The decentralization policy allows local government to make new policies in order to improve the economy, which led to large expansion of oil palm plantations in the Riau Province.

### Tropical Rainforest Cover Lost

Tropical rainforest cover decreased from ∼63% in the period 1990s to ∼37% in the 2000s with a notable decline in intact forest area. In 2012, the Riau Province’s tropical rainforest covered only ∼22%. In the 1990s, the major proximate causes of tropical rainforest cover loss were attributed to a transmigration program, which caused tropical rainforest to be transformed into settlements, land clearing activity for oil palm and rubber plantation, and the use of fires for agricultural conversion. In the Soeharto era, there was a five-year economic development plan (Repelita Program). The Repelita III fiscal year (FY) 1979–1984 emphasized development of agriculture-related industry and other industries. During this period, the Central Government made 591,000 ha of forests available for conversion, among others 110,380 ha for plantations and 124,980 ha for transmigrant resettlements. During the Repelita IV period (FY 1984–1989), 600,000 ha of existing forests were converted into oil palm and rubber plantation, in addition to land converted for transmigration. The Repelita V period (FY) 1989–1994 targeted transportation and communications. During the 1980s, these plans also gave a greater role for private capital in industry. In the 2000s up to 2012, the loss of tropical rainforest corresponded with higher prices of world palm oil and higher demand from European countries [21]. Cultivated areas were always increasing during the periods of analysis, as the main livelihood of the local people of the Riau province is shifted to cultivation of crops. The cultivated area increased from ∼23% of the total provincial area in 1990s to ∼37% in the 2000s and ∼46% in 2012.

**Figure 3:**
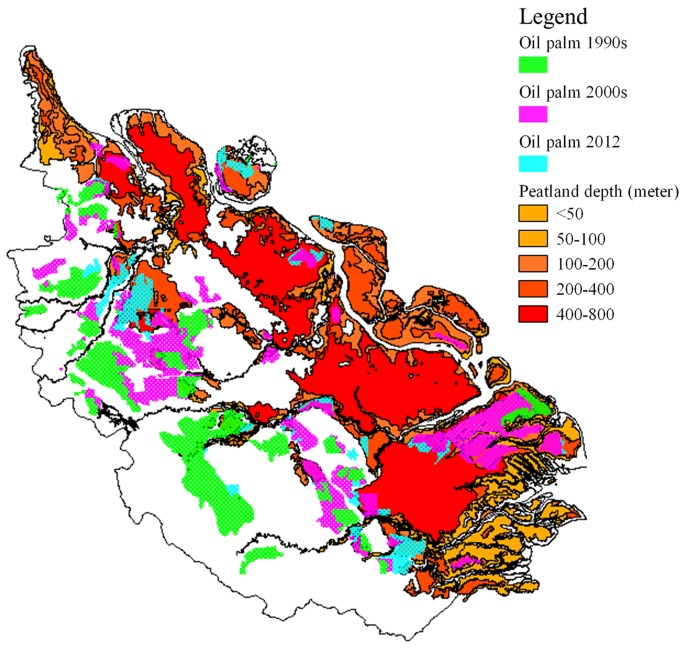
Oil palm development on peatlands.

Figure 3 shows that oil palm plantation first were converted from tropical forest at the Western side of the Riau Province, then gradually expanding to the Eastern side. The Western side land cover originally was tropical forest and the Eastern side was peatlands with varying depth.

### CO_2_ Emission from Peatlands Drained for Oil Palm Plantations

In the 1990s and 2000s, total CO_2_ released to the atmosphere was estimated at ∼538,000 tCO_2_.y^−1^, with peatlands as the source of 40.62% CO_2_ emissions in this period. After 2000s up to 2012, total CO_2_ emitted to the atmosphere was estimated at ∼246,000 tCO_2_.y^−1^ (Table 2), with 69.94% of the CO_2_ emissions generated from converted peatlands.

**Table 2:** Total CO_2_ emitted from drained peatlands.

	1990s–2000s	2000s–2012
**Land occupation growth (ha)**	354,123.83	162,004.84
**Carbon loss (tC.y^-1^)**	146,607.26	67,070.00
**CO_2_ emitted (tCO_2_.y^-1^)**	538,048.66	246,146.92

### CO_2_ Emission from Forest Converted for Oil Palm Plantations

In the 1990s and 2000s, total CO_2_ released to the atmosphere was estimated at ∼26 million tCO_2_.y^−1^, and forests were the source of 59.38% of the CO_2_ emissions in this period. After 2000s up to 2012, total CO_2_ released to the atmosphere was estimated at ∼4.9 million tCO_2_.y^−1^ (Table 3), where 27.62% of the CO_2_ emissions were generated from converted forest. This data infers that in the Riau Province, the oil palm industry boomed in this period.

**Table 3:** Total CO_2_ emitted from forest conversion.

	1990s–2000s	2000s–2012
**Land occupation growth (ha)**	355,236.32	67,877.77
**Carbon loss (tC.y^-1^)**	7,014,726.46	1,357,555.40
**CO_2_ emitted (tCO_2_.y^-1^)**	26,074,346.11	4,982,228.32

## Conclusions

Results obtained in this study show that in the Riau Province, the palm oil industry boomed between the 1990s and 2000s period, with tropical forests and peat land as the primary land source for new plantations. Tropical forest was the primary land source in the period 1990s-2000s, while expansion in deeper peatlands started after 2000s to 2012. Decreasing of CO_2_ emissions in the 2000s up to 2012 was possibly due to the enforcement of the new policy i.e. a moratorium on forest conversion, which meant that the granting of new permits/leases, forest use, forest use change, and other use areas was delayed [22].

Our study documentes the basic expansion pattern of oil palm production, from the Western side of the province to the Eastern side. Our result suggests that the expansion of oil palm plantations occurred in peatlands in the period 1990s–2000s and generated above ground biomass loss (∼40%). The carbon emission continued to increase during the period of 2000s–2012 (∼70% generated from peatlands). This finding implies that the palm oil industry was the main perpetrator of tropical forest deforestation at provincial level in the early stages. Furthermore, the expansion using the peatlands was applied in the next stage of development. Development of oil palm plantation in mangrove forest was also noted, although the conversion area was only small.

Approximately 5% of global and 50% of tropical peatlands are located in Indonesia [23], [24]. CO_2_ emissions from conversion of peatland are a unique challenge for Indonesia, as they account for 58% of global emissions from peat decomposition [25]. However, results suggested that in recent times, the moratorium on deforestation has played a significant role in decelerating the development of oil palm plantations in this leading province.
